# Transcriptomic profiling and targeted validation reveal molecular mechanisms of oxygen therapy in high-altitude cerebral injury

**DOI:** 10.3389/fnins.2026.1738756

**Published:** 2026-04-13

**Authors:** Xiaojie Hu, Xuedong Bai, Shuyi Pan, Hang Li

**Affiliations:** 1 Department of Hyperbaric Oxygen, Sixth Medical Center, Chinese PLA General Hospital, Beijing, China; 2 School of Medicine, South China University of Technology, Guangzhou, China; 3 Department of Orthopedics, Sixth Medical Center, Chinese PLA General Hospital, Beijing, China

**Keywords:** high-altitude cerebral injury, hyperbaric oxygen, neuroinflammation, oxidative stress, PI3K–AKT pathway, TLR4–NF-κB pathway, transcriptomics

## Abstract

**Background:**

Exposure to high-altitude hypoxia is associated with an increased risk of impaired brain structure and function, with oxidative stress and neuroinflammation widely recognized as key mechanisms involved. In this context, hyperbaric oxygen therapy is considered a potential intervention; however, the mechanism by which it affects cerebral function changes caused by high-altitude exposure remains to be further elucidated.

**Objective:**

This study aims to explore and compare the therapeutic effects of normobaric oxygen (NBO) and hyperbaric oxygen (HBO) on high-altitude cerebral injury (HACI), and to elucidate the molecular mechanisms underlying their neuroprotective effects using transcriptomic profiling and targeted validation.

**Methods:**

A mouse model of high-altitude cerebral injury was established using a hypobaric hypoxia chamber. Mice were exposed to a simulated altitude of 7,000 m (approximately 9.8% O₂ at 0.47 ATA) for 3 consecutive days to induce severe hypoxia. Animals were divided into four groups: Control (Con), High-Altitude exposure (HH), post-HH treated with normobaric oxygen (NBO; 100% O₂ at 1.0 ATA for 1 h daily for 3 days), and post-HH treated with hyperbaric oxygen (HBO; 100% O₂ at 2.0 ATA for 1 h daily for 3 days). Brain tissues were analyzed using H&E staining, RNA sequencing (RNA-seq), Western blotting for key pathway proteins, immunofluorescence for glial cell activation, and ELISA for inflammatory cytokines. Oxidative stress markers (SOD, MDA, GSH, NO) were also assessed.

**Results:**

Histopathological analysis confirmed cerebral damage in the HH group, which was significantly ameliorated by both HBO and NBO treatments. RNA-seq revealed widespread disruption of the cerebral transcriptome following high-altitude exposure. Oxygen therapy was associated with partial restoration of global gene expression patterns. KEGG pathway analysis highlighted significant enrichment in pathways related to NF-κB signaling, cytokine–cytokine receptor interaction, IL-17 signaling, and PI3K–AKT signaling. Subsequent targeted validation demonstrated that oxygen treatment reduced oxidative stress (increased SOD and GSH; decreased MDA and NO) and modulated the PI3K–AKT signaling pathway (increased p-AKT/AKT). Concurrently, oxygen therapy attenuated neuroinflammatory responses, inhibiting microglial and astrocytic activation, reducing pro-inflammatory cytokine levels (IL-1β, IL-6, TNF-α), and modulating the TLR4–NF-κB signaling axis (decreased TLR4 and p-p65/p65). HBO treatment was associated with broader modulation of several molecular pathways involved in oxidative stress and inflammation.

**Conclusion:**

Existing evidence suggests that HBO may exert protective effects against altitude-related brain injury. This mechanism likely involves activating the PI3K–AKT/Nrf2 axis to alleviate oxidative stress and inhibiting the TLR4–NF-κB pathway to reduce neuroinflammation, thereby partially restoring transcriptional homeostasis. However, the causal relationships between these pathways and their interactions require further validation and refinement.

## 1 Introduction

High-altitude environments, characterized by hypobaric hypoxia, pose significant challenges to human physiology, with the brain being particularly vulnerable due to its high metabolic demand and oxygen dependence (Fabries et al., 2025). High-altitude cerebral injury (HACI) typically occurs at altitudes above 2,500 m, encompassing conditions ranging from acute mountain sickness (AMS) to more severe outcomes such as high-altitude cerebral edema (HACE) (Gonggalanzi et al., 2016; Luks and Hackett, 2022). Exposure to extremely high altitudes (e.g., ≥7,000 m, ~9.8% O_2_), as modeled in this study (Burtscher et al., 2018; Netzer et al., 2013), poses substantially greater hypoxic stress, which can overwhelm adaptive mechanisms in the central nervous system. It is important to distinguish such extreme altitudes from moderate altitudes, where adaptive responses can partially preserve cerebral homeostasis. Available evidence suggests that the occurrence of HACI involves multiple interacting pathological processes, in which aggravated oxidative stress and neuroinflammation have been repeatedly reported and are considered to be one of the key mechanisms involved (Shushanyan et al., 2024; Wang et al., 2022). The drastic reduction in partial pressure of oxygen at high altitude disrupts mitochondrial electron transport chains, leading to excessive generation of reactive oxygen species (ROS) and reactive nitrogen species (RNS) (Lira-Mejía et al., 2025). This oxidative burden overwhelms endogenous antioxidant defenses, resulting in lipid peroxidation, protein oxidation, and DNA damage, ultimately triggering neuronal apoptosis and dysfunction (Zhang X. et al., 2025).

Concurrently, hypoxic stress activates the brain’s innate immune response, predominantly mediated by microglia and astrocytes (Johnson et al., 2007). This activation instigates a robust neuroinflammatory cascade, characterized by the release of pro-inflammatory cytokines such as interleukin-1β (IL-1β), interleukin-6 (IL-6), and tumor necrosis factor-α (TNF-α) (Chen et al., 2025). Key signaling pathways like the Toll-like receptor 4/nuclear factor-kappa B (TLR4–NF-κB) pathway are pivotal amplifiers of this inflammatory response, perpetuating a cytotoxic environment that exacerbates neuronal damage (Liu S. et al., 2022; Tang et al., 2019). Conversely, endogenous protective pathways exist to counteract these deleterious processes. The nuclear factor erythroid 2-related factor 2 (Nrf2) pathway is a master regulator of cellular antioxidant responses, orchestrating the expression of cytoprotective genes like heme oxygenase-1 (HO-1) (Osburn and Kensler, 2008; Wang et al., 2023). Similarly, the phosphatidylinositol 3-kinase/protein kinase B (PI3K–AKT) pathway promotes cell survival, growth, and metabolism, and its activation has been shown to confer neuroprotection in various injury models, partly through cross-talk with Nrf2 and inhibition of inflammatory signals (El-Samad et al., 2025; Huang et al., 2025). The intricate balance between these damaging (TLR4–NF-κB) and protective (Nrf2, PI3K–AKT) pathways ultimately determines the extent of cerebral injury.

Hyperbaric oxygen (HBO) therapy, involving the inhalation of 100% oxygen at pressures greater than one atmosphere absolute (ATA), has been explored as a potential treatment for hypoxic–ischemic conditions (Hajek et al., 2025). HBO is believed to alleviate hypoxic load, reduce brain edema and regulate inflammatory responses by significantly enhancing the ability of tissue oxygen delivery. Some studies also suggest its clinical or preclinical benefits in neurological diseases (Xue et al., 2023). However, the mechanism of HBO in HACI has not been systematically elucidated, especially the lack of direct evidence about its effects on the whole genome expression profile and whether and how it targets key pathways such as oxidative stress and neuroinflammation (Bin-Alamer et al., 2024). While more readily available, the efficacy of normobaric oxygen (NBO) is generally considered to be lower than HBO, and comparative studies between the two are critical to understanding the contribution of “stress factors” to efficacy. Therefore, it is necessary to develop a comprehensive research framework with transcriptomics as the core and mechanism verification: characterize the molecular remodeling trajectory of HBO/NBO in hypoxic brain tissue through whole genome expression analysis, supplemented by genetic/pharmacological intervention and phenotypic evaluation, to verify the causal role and necessity of these pathways. This strategy is expected to not only elucidate the molecular basis of HBO’s improvement of high-altitude cerebral injury and clarify the role of stress parameters in the formation of efficacy, but also provide mechanistic basis and biomarker clues for the development of dose-stress-time optimization and higher quality translational and clinical studies.

This study was designed to first utilize RNA sequencing to map the global transcriptomic alterations induced by high-altitude exposure and to identify which pathways are most effectively reversed by HBO therapy. We then employed targeted molecular biology techniques—Western blotting, immunofluorescence, ELISA, and oxidative stress assays—to rigorously validate the core pathways identified by the transcriptomic analysis. This strategy, moving from systemic discovery to focused validation, aims to provide a comprehensive and mechanistic elucidation of HBO’s therapeutic potential against HACI, potentially revealing novel targets for intervention.

## 2 Materials and methods

### 2.1 Animal model and grouping

Nine-week-old male C57BL/6 mice (weighing 20 ± 2 g at the start of the experiment) were housed under controlled conditions (22 ± 1 °C, 12 h/12 h light/dark cycle) with free access to food and water. After all treatments were completed, the mice were intraperitoneally injected with sodium pentobarbital (Sigma-Aldrich, USA; Cat. No. 4390-16-3) at a dose of 150 mg/kg of body weight. Tissue collection was then performed immediately following confirmation of death to minimize tissue degradation. All experimental protocols were approved by the Institutional Animal Experiment Committee of the Sixth Medical Center, PLA General Hospital and were conducted in accordance with the Regulations for the Administration of Affairs Concerning Experimental Animals (China) (only male mice were used in this initial study to avoid potential confounding effects of the estrous cycle on neuroinflammatory and oxidative stress responses, thereby reducing within-group variability for mechanistic exploration).

The animals were randomly assigned to one of the following four groups:

Control (Con): Mice were maintained under normobaric normoxic conditions (21% O₂ at 1.0 ATA) throughout the experimental period.

High-altitude Hypoxia (HH): Mice were exposed to a simulated high altitude of 7,000 m (approximately 9.8% O_2_ at 0.47 ATA) for 3 days, with food and water provided ad libitum. After hypoxic exposure, HH mice were maintained under normobaric air for 3 days, with food and water provided ad libitum, and without any additional treatment.

HH + NBO: Following 3 days of hypoxic exposure, mice received normobaric hyperoxia therapy (NBO: 100% O₂ at 1.0 ATA for 1 h per day at a constant inflow and outflow rate to maintain stable chamber pressure and prevent CO₂ accumulation) for three consecutive days with food and water provided ad libitum.

HH + HBO: After 3 days of hypoxic exposure, mice were treated with hyperbaric oxygen (HBO: 100% O₂ at 2.0 ATA for 1 h daily) for three consecutive days with food and water provided ad libitum. The chamber was continuously flushed with 100% oxygen at a flow rate of 5 L/min.

**Figure 1:**
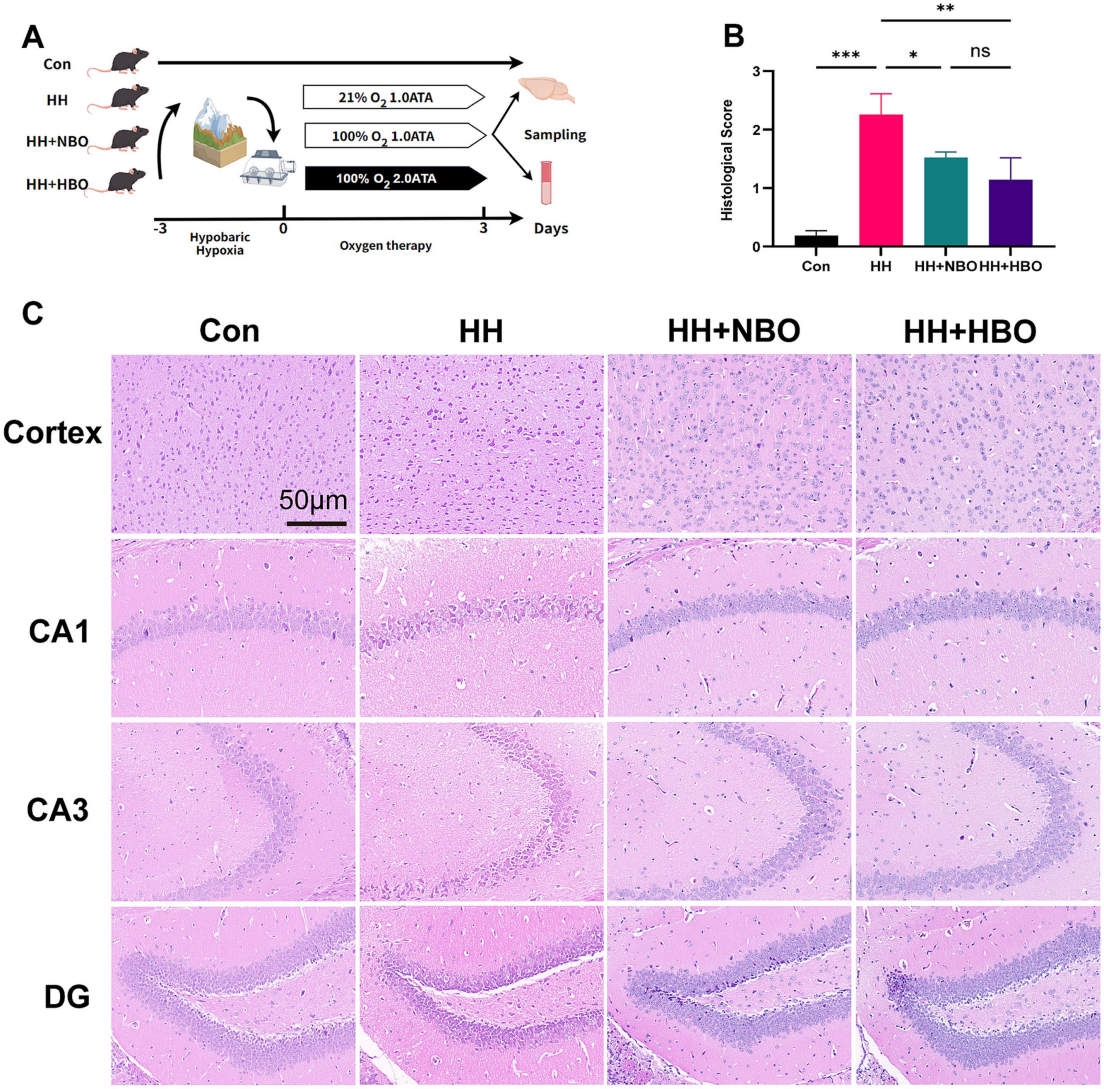
Hyperbaric and normobaric oxygen therapies significantly ameliorate acute hypobaric hypoxia-induced neural damage. **(A)** Schematic illustration of the experimental timeline. Mice were exposed to hypobaric hypoxia for 3 days followed immediately by 3 days of oxygen treatment (NBO or HBO). Brain tissues were collected on day 3 for pathological evaluation. **(B)** Quantitative analysis of pathological scores in cortical and hippocampal regions. Data are presented as mean ± SD (*n* = 3 mice per group, with 3 fields analyzed per mouse). Statistical significance was determined by one-way ANOVA followed by Tukey’s *post-hoc* test. *p < 0.05, **p < 0.01, ***p < 0.001; ns, not significant. **(C)** Representative H&E-stained images showing morphological features in the cerebral cortex, hippocampal CA1, CA3, and dentate gyrus (DG) regions from all four experimental groups. The HH group exhibits severe neuronal damage evidenced by pyknotic nuclei and vacuolization. These pathological alterations are substantially reduced in both oxygen-treated groups, with the HBO group showing the most pronounced preservation of normal cytoarchitecture. Scale bar, 50 μm.

Decompression was performed uniformly at a rate of 0.2 kg/cm^2^ per minute. The high and low pressure oxygen chamber was provided by the Hyperbaric Oxygen Department of the Sixth Medical Center of the PLA General Hospital. The experimental procedure is shown in Figure 1A.

### 2.2 Histopathological assessment (H&E staining)

After the animals were sacrificed, the mouse brains were isolated and perfused transcardially with ice-cold PBS followed by fixation via immersion in freshly prepared 4% paraformaldehyde (PFA) in 0.1 M phosphate buffer (pH 7.4) at 4 °C for 24 h, as per established protocols (Wu et al., 2021). Fixed brains were then embedded in paraffin and sectioned at 5 μm thickness. Tissue sections were dewaxed and stained with hematoxylin–eosin (H&E) following standard procedures. Histopathological changes were examined under a Nikon Fi3 microscope (Nikon, Japan) at 40× magnification. Pathological scoring was performed by two professional histologists blinded to the experimental groups, using a semi-quantitative scale (0–4) based on neuronal damage: 0, no damage (intact tissue structure); 1, mild injury (sporadic cavitation); 2, moderate injury (nuclear consolidation with cavitation); 3, severe injury (extensive nuclear shrinkage and structural failure); 4, extremely severe injury (widespread necrosis).

### 2.3 RNA sequencing and bioinformatic analysis

Total RNA was extracted from brain tissues using TRIzol Reagent (Thermo Fisher Scientific, USA, Cat. No. 15596026). RNA quality was assessed with a NanoDrop spectrophotometer (Thermo Fisher Scientific) and an Agilent 2100 Bioanalyzer (Agilent Technologies); all samples had an RNA Integrity Number (RIN) ≥ 7. Sequencing libraries were prepared by Shanghai Personal Biotechnology Cp. Ltd., including mRNA enrichment, cDNA synthesis, adapter ligation, and size selection using AMPure XP beads (Beckman Coulter, Cat. No. A63881). Libraries were quantified with the Agilent High Sensitivity DNA Kit (Agilent Technologies, Cat. No. 5067-4626) and sequenced on an Illumina NovaSeq 6000 platform to generate 150-bp paired-end reads. Raw reads were processed with fastp (v0.22.0) for adapter trimming and quality filtering. Clean reads were aligned to the mouse genome (GRCm38) using HISAT2 (v2.1.0). Gene expression levels were quantified using raw read counts generated by HTSeq (v0.9.1). Differential expression analysis was performed with DESeq2. Genes with |log_2_ fold change| > 1 and *p*-value < 0.05 were considered differentially expressed. Gene Ontology (GO) and Kyoto Encyclopedia of Genes and Genomes (KEGG) pathway enrichment analyses of the differentially expressed genes were conducted using the online platform https://www.genescloud.cn/home.

### 2.4 Biochemical analysis

The brain tissues were harvested from mice following anesthesia-induced sacrifice, then homogenized in pre-cooled 0.9% saline solution and centrifuged at 3,500 rpm for 10 min at 4 °C. The resulting supernatants were collected and assayed for malondialdehyde (MDA), glutathione (GSH), superoxide dismutase (SOD), and nitric oxide (NO) using commercially available assay kits (Jiancheng Institute of Bioengineering Co., Ltd., Nanjing, China). Protein concentration was determined using the bicinchoninic acid (BCA) method (Keygen Biotech, Nanjing, China).

### 2.5 Western blotting

Proteins were extracted from hippocampal tissues using ice-cold RIPA lysis buffer. After centrifugation at 12,000 × g for 10 min at 4 °C, the supernatant containing total protein was collected and the loading volume was calculated. The proteins were denatured and separated by SDS–polyacrylamide gel electrophoresis (SDS–PAGE) and then electrophoretically transferred onto polyvinylidene fluoride (PVDF) membranes. The membrane was blocked with 5% skimmed milk in TBST for 1 h at room temperature, and then washed with TBST. Primary antibodies, including p-PI3K (1:1,000, bs-6417R, Bioss, USA), p-AKT (1:1,000, bs-0876R, Bioss, USA), Nrf-2 (nuclear) (1:1,000, bs-1074R, Bioss, USA), HO-1 (1:2,000, ab189491, Abcam, UK), TLR4 (1:1,000, 19811-1-AP, Proteintech, China), p-p65 (1:1,000, AF2006, Affinity, USA), PI3K (1:25,000, 60225-1-Ig, Proteintech, China), p65 (1:3,000, 10745-1-AP, Proteintech, China), AKT (1:25,000, 60203-2-Ig, Proteintech, China), and GAPDH (1:15,000, ab181602, Abcam, UK), were added and incubated at 4 °C overnight. After washing with TBST, horseradish peroxidase-conjugated goat anti-rabbit IgG secondary antibody (1:5,000, SA00001-2, Proteintech, China) or goat anti-mouse IgG secondary antibody (1:5,000, SA00001-1, Proteintech, China) was added and incubated at room temperature for 2 h. All antibodies were diluted with antibody diluent (P0023A, Beyotime, China). The membrane was then incubated with a supersensitive ECL chemiluminescent substrate kit (Thermo Fisher, USA) and exposed in a gel imager. The relative gray values of the protein bands were analyzed using Image Lab software.

### 2.6 Immunofluorescence and immunohistochemical staining

Mice were anesthetized and perfused with PBS followed by 4% paraformaldehyde through the left cardiac ventricle. The brains were extracted and fixed in 4% paraformaldehyde at 4 °C for 24 h, then immersed in phosphate buffer containing 20% or 30% sucrose. The tissues were subsequently embedded in OCT and sliced into 10-μm-thick sections using a cryostat.

For immunofluorescence staining, sections were rinsed with PBS for 15 min and blocked with 1% bovine serum albumin (BSA) in 0.3% Triton X-100 for 1 h. Sections were then incubated overnight at 4 °C with primary antibodies against NeuN (1:400, 26975-1-AP, Proteintech, Wuhan, China), IBA-1 (1:200, 10904-1-AP, Proteintech, Wuhan, China), and GFAP (1:200, 16825-1-AP, Proteintech, Wuhan, China). After washing with PBS, sections were incubated with appropriate fluorescent secondary antibodies for 1 h at room temperature in the dark. Nuclei were counterstained with DAPI. Images were captured using a laser confocal microscope (ZEISS LSM 880, Carl Zeiss AG, Germany).

For immunohistochemistry, inflammation was evaluated using an anti-IL-6 antibody (1:100, Proteintech, Wuhan, China). Sections were incubated with the primary antibody followed by HRP-conjugated secondary antibody and visualized using DAB substrate according to the manufacturer’s protocol. Images were captured using a light microscope.

### 2.7 Enzyme-linked immunosorbent assay (ELISA)

Blood was collected via cardiac puncture into serum separator tubes without anticoagulant. Serum samples were separated from the collected blood through centrifugation (3,000 × g, 10 min, 4 °C). According to the manufacturer’s instructions, the levels of interleukin (IL)-1β, IL-6, and tumor necrosis factor (TNF)-α in the serum were determined using enzyme-linked immunosorbent assay (ELISA) kits (JL18442, JL20268, and JL10484; JONLNBIO, Shanghai, China). Serum samples were diluted 1:5 using the dilution buffer provided in the kits, and all samples were assayed in duplicate.

### 2.8 Statistical analysis

GraphPad Prism 10 was used to conduct the statistical analysis. Data are expressed as the mean ± standard deviation (SD) of at least three independent experiments unless otherwise indicated. One-way ANOVA and Tukey’s test were used for comparisons between multiple groups, and two-tailed Student’s *t*-test for used for comparisons between two groups. *p*-values < 0.05 were considered statistically significant.

## 3 Results

### 3.1 HBO alleviates neuropathological impairments induced by high-altitude exposure

Acute exposure to hypobaric hypoxia can induce significant histopathological alterations in the brains of mice. As illustrated in Figures 1B,C, the histopathological score in the untreated hypobaric hypoxia exposure (HH) group was markedly elevated compared to that in the control (Con) group (*p* < 0.001), with prominent features including cytoplasmic vacuolation, nuclear condensation, and structural damage within the cortical and hippocampal regions. Both oxygen treatment groups showed partial improvement in histological appearance relative to the HH group. In comparison to the HH group, the histopathological score in the hyperbaric oxygen (HBO) group was significantly reduced (*p* < 0.01). Normobaric oxygen (NBO) treatment also led to a significant decrease in histopathological damage relative to the HH group (*p* < 0.05). Although the HBO group exhibited numerically lower injury scores than the NBO group, the difference between these two treatments did not reach statistical significance (*p* > 0.05). These findings suggest that both normobaric and hyperbaric oxygen therapy administered for three consecutive sessions post-hypoxia exposure exerted significant neuroprotective effects, effectively alleviating cellular edema and pericellular space expansion induced by hypobaric hypoxia. In addition, histological scores in both treatment groups remained higher than those in the control group, indicating that oxygen intervention attenuated but did not completely reverse hypoxia-induced neuronal injury.

### 3.2 Transcriptomic profiling reveals a systemic restoration of gene expression networks by HBO

#### 3.2.1 Identification of DEGs

**Figure 2:**
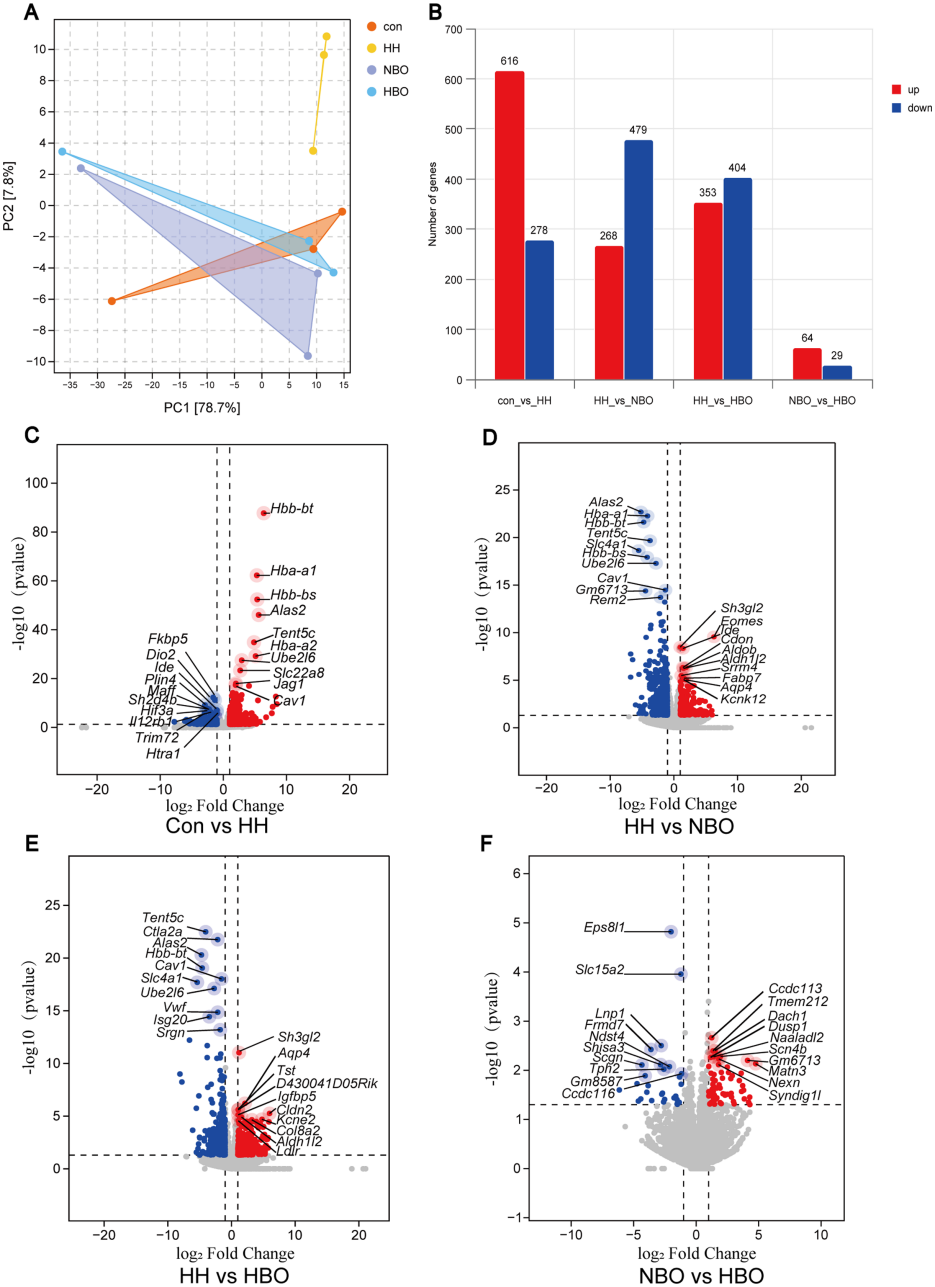
Transcriptomic profiling reveals distinct gene expression patterns and broader molecular modulation associated with hyperbaric oxygen treatment. **(A)** Principal component analysis (PCA) plot of transcriptomes from all four experimental groups. **(B)** Bar plot displaying the number of significantly upregulated and downregulated differentially expressed genes (DEGs) (FDR < 0.05, |log_2_FC| > 1) for the key pairwise comparisons: Con vs. HH, HH vs. HH + NBO, and HH vs. HH + HBO, NBO vs. HBO. HBO treatment induces the most significant reversal of HH-induced transcriptional changes. **(C–F)** Volcano plots visualizing the differential gene expression for the pairwise comparisons: **(C)** Con vs. HH, **(D)** HH vs. HH + NBO, **(E)** HH vs. HH + HBO, **(F)** NBO vs. HBO. Genes with no significant change are shown in gray. Significantly upregulated (red) and downregulated (blue) genes are highlighted (FDR < 0.05). All pairwise comparisons of the differentially expressed genes are defined as “Reference group vs. Experimental group,” and the upregulation/downregulation refers to the expression change of the experimental group relative to the reference group. The top 10 most significantly upregulated and downregulated genes in each comparison are explicitly labeled with their gene symbols (*n* = 3 mice per group).

Unbiased transcriptomic analysis by RNA-seq was employed to investigate the global genomic response. Principal component analysis (PCA) demonstrated a clear separation between the Con and HH groups along principal component 1 (PC1), which accounts for 78.7% of the total variance (Figure 2A). This suggests that high-altitude exposure induced substantial alterations in the transcriptome. Both oxygen therapy groups exhibited deviations from the HH group’s trajectory, with the HBO group showing the closest proximity to the control group, suggesting a more effective restoration of normal transcriptional states. This observation was corroborated by differential gene expression analysis. A comparison between the HH group and the Control group revealed significant dysregulation of the 894 gene, with 616 being upregulated and 278 being downregulated (criteria: log2FC > 1, adj. *p* < 0.05, same as other groups). Between the HH and NBO groups, 747 DEGs were obtained, including 479 downregulated genes and 268 upregulated genes. Moreover, 757 DEGs were detected between the HH group and the HBO group, consisting of 404 downregulated genes and 353 upregulated genes (Figure 2B). It indicates that HBO treatment has a powerful reversing effect on gene expression changes, highlighting its extensive regulatory role. We visually summarized the differential gene expression profiles obtained using Volcano plots (Figures 2C–F), in which the top 10 genes with the most significant up-regulation and down-regulation were clearly labeled. The comparison between Control and HH groups (Figure 2C) revealed a robust adaptive response to hypoxia, primarily characterized by the marked upregulation of erythropoiesis-related genes such as *Hba-a1*, *Hbb-bt,* and *Alas2*, aimed at enhancing oxygen transport capacity. Concurrently, a downregulation of genes involved in stress response and immunomodulation (*Fkbp5, Dio2*) was observed, suggesting a suppression of non-essential pathways to conserve energy.

Notably, the comparison between HH and NBO treatments indicated that NBO only partially reversed this HH-induced transcriptional signature (Figure 2D). While it effectively downregulated key erythroid genes (*Alas2*, *Hba-a1*), its modulatory effect on the heightened inflammatory and interferon-response pathways (*Ube2l6*, *Isg20*) was limited.

In stark contrast, the HH vs. HBO comparison demonstrated a far more comprehensive restorative effect (Figure 2E). HBO treatment not only normalized the expression of core erythropoietic genes but also significantly suppressed pivotal mediators of the hyperinflammatory and interferon response (*Ube2l6*, *Isg20*, *Srgn*). Furthermore, HBO increased the expression of *Aqp4*, a gene involved in water homeostasis and closely associated with neurovascular unit function, suggesting a potential role in maintaining neurovascular stability.

The direct comparison between the two oxygen therapies (NBO vs. HBO) unveiled fundamentally distinct transcriptional programs (Figure 2F). HBO uniquely regulated pathways related to tissue remodeling and TGF-β signaling (*Ccdc113*), highlighting a mechanism of action that extends beyond mere oxygen supplementation.

In summary, transcriptomic profiling revealed that both oxygen therapies influenced multiple biological processes associated with hypobaric hypoxia. Compared with NBO, HBO treatment showed broader modulation of genes involved in metabolic adaptation, inflammatory regulation, and vascular homeostasis. These findings provide additional insight into the molecular responses to oxygen therapy in high-altitude cerebral injury.

#### 3.2.2 KEGG and GO enrichment analyses of the DEGs

**Figure 3:**
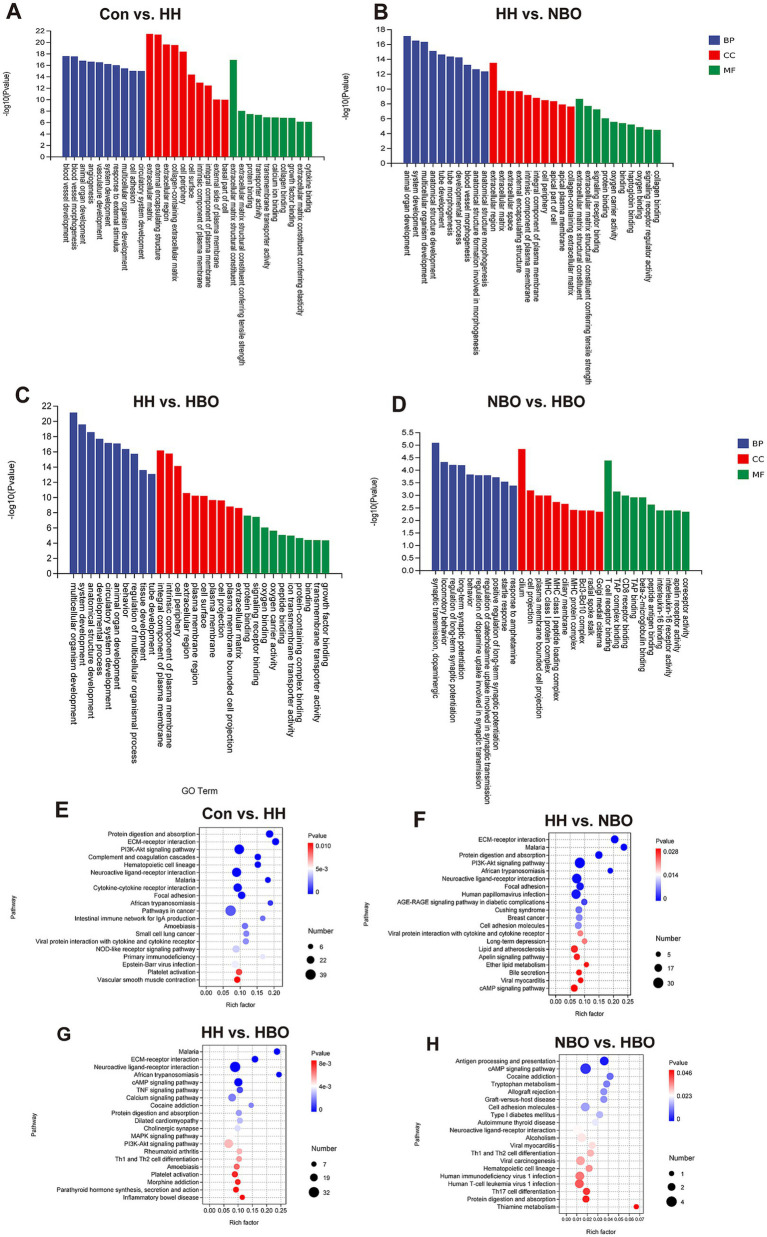
Functional enrichment analysis of differentially expressed genes across comparison groups. **(A–D)** Gene ONTOLOGY (GO) enrichment analysis of biological processes for the following comparisons: **(A)** Con vs. HH, **(B)** HH vs. NBO, **(C)** HH vs. HBO, and **(D)** NBO vs. HBO. The top 10 significantly enriched terms (FDR < 0.05) are shown for each comparison, demonstrating distinct patterns of biological processes affected by hypobaric hypoxia and modulated by oxygen therapies. **(E–H)** Kyoto Encyclopedia of Genes and Genomes (KEGG) pathway enrichment analysis for the same comparisons: **(E)** Con vs. HH, **(F)** HH vs. NBO, **(G)** HH vs. HBO, and **(H)** NBO vs. HBO. The top 20 significantly enriched pathways (FDR < 0.05) are displayed, highlighting key signaling pathways involved in the response to hypobaric hypoxia and the differential effects of oxygen interventions. Dot size represents the number of genes enriched in each term/pathway, and color intensity indicates the statistical significance (−log10(FDR)). Notable pathways discussed in the text are labeled (*n* = 3 per group).

To elucidate the biological processes and pathways involved in hypobaric hypoxia-induced cerebral injury and the therapeutic mechanisms of oxygen interventions, we performed Gene Ontology (GO) and Kyoto Encyclopedia of Genes and Genomes (KEGG) pathway enrichment analyses on differentially expressed genes (DEGs) from each comparison group (Figure 3).

In the comparison between control and hypobaric hypoxia-exposed groups (Con vs. HH), GO analysis revealed significant enrichment of terms associated with vascular development and morphogenesis (e.g., blood vessel development, angiogenesis), extracellular matrix (ECM) organization, and response to external stimuli (Figure 3A; Supplementary Table 1). These enrichment results are suggestive of the biological processes impacted by hypoxia. Concurrently, KEGG pathway analysis indicated activation potential activation of pathways related to ECM-receptor interaction, PI3K–Akt signaling, complement and coagulation cascades, and cytokine-cytokine receptor interaction (Figure 3E; Supplementary Table 5), reflecting pronounced vascular remodeling and inflammatory responses that may occur following acute hypobaric hypoxia exposure.

When comparing hypobaric hypoxia-exposed groups with and without normobaric oxygen treatment (HH vs. NBO), GO terms remained enriched in developmental and circulatory system processes (Figure 3B; Supplementary Table 2), with persistent activation of pathways such as ECM-receptor interaction, TNF signaling, and neuroactive ligand-receptor interaction (Figure 3F; Supplementary Table 6), suggesting that NBO may have a limited modulating effect on hypoxia-induced responses.

In contrast, hyperbaric oxygen treatment (HH vs. HBO) resulted in a distinct enrichment profile, with GO terms highlighting processes involved in tissue morphogenesis and ECM structural organization (Figure 3C; Supplementary Table 3). KEGG analysis showed that HBO was associated with significant modulation of pathways including ECM-receptor interaction, focal adhesion, AGE-RAGE signaling in diabetic complications, and apelin signaling (Figure 3G; Supplementary Table 7), indicating a potential restorative effect on vascular integrity and attenuation of inflammatory and stress-related pathways.

Notably, direct comparison between the two oxygen therapies (NBO vs. HBO) revealed differences in several biological processes and signaling pathways. GO analysis identified enrichment in terms related to synaptic transmission and behavior (Figure 3D; Supplementary Table 4). KEGG analysis further highlighted differential enrichment of immune- and metabolism-related pathways, including antigen processing and presentation, cAMP signaling, and tryptophan metabolism (Figure 3H; Supplementary Table 8), suggesting that HBO may induce broader modulation of certain hypoxia-related molecular pathways.

### 3.3 HBO mitigates oxidative stress by activating the PI3K–AKT pathways

**Figure 4:**
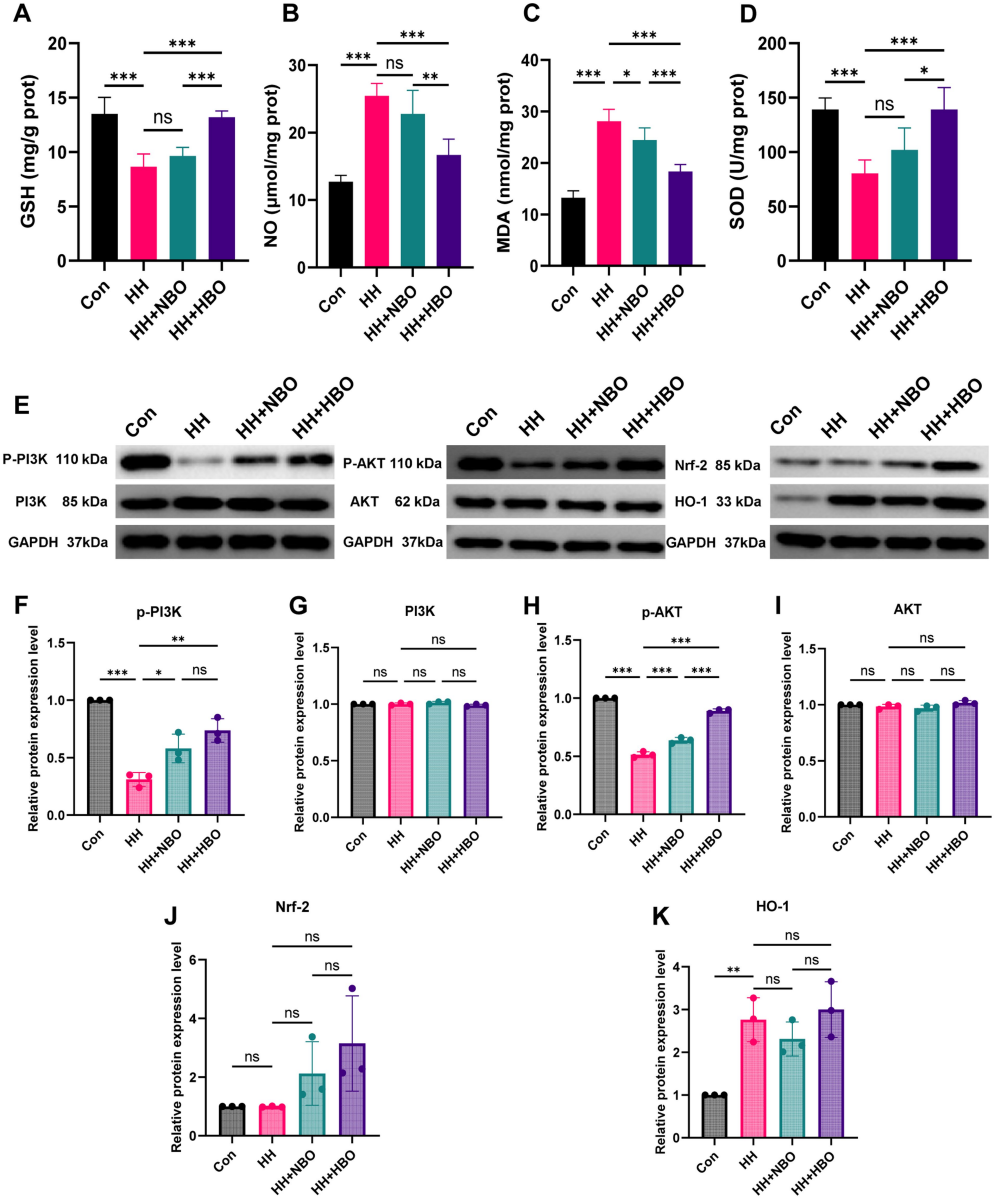
Functional oxidative stress parameters and activation of related signaling pathways in mouse brain tissue after hypobaric hypoxia exposure and oxygen therapies. **(A–D)** Assessment of oxidative stress levels. **(A)** Glutathione (GSH) levels. **(B)** Nitric oxide (NO) levels. **(C)** Malondialdehyde (MDA) levels. **(D)** Superoxide dismutase (SOD) activity. **(E)** Representative western blot images of key proteins in the PI3K–Akt and Nrf-2/HO-1 pathways, including p-PI3K, PI3K, p-AKT, AKT, Nrf2, and HO-1. **(F–K)** Densitometric quantification of western blot results normalized to loading control GAPDH protein. **(F)** p-PI3K protein levels. **(G)** p-AKT protein levels. **(H)** Total PI3K protein levels. **(I)** Total AKT protein levels. **(J)** Nrf2 protein levels. **(K)** HO-1 protein levels. Data are presented as mean ± SD (*n* = 5 per group), *p < 0.05, **p < 0.01, ***p < 0.001 by one-way ANOVA followed by Tukey’s *post-hoc* test.

Guided by the transcriptomic findings highlighting oxidative stress and PI3K–AKT pathways, we first assessed functional oxidative stress parameters (Figures 4A–D). The HH group exhibited a significant oxidative imbalance: decreased activities of the antioxidant enzymes SOD (*p* < 0.001 vs. Con) and GSH (*p* < 0.001 vs. Con), alongside increased levels of the lipid peroxidation product MDA (*p* < 0.001 vs. Con) and nitric oxide (NO) (*p* < 0.001 vs. Con). HBO treatment most effectively reversed these changes, significantly increasing SOD and GSH levels and decreasing MDA and NO levels compared to the HH group (*p* < 0.001). The effects of NBO were significant but weaker than HBO for SOD and MDA.

To explore the molecular mechanism behind this functional improvement, we performed Western blot analysis to verify its protein-level activation and to explore its role in different oxygen therapies. The HH group exhibited significant inhibition of the PI3K–Akt pathway, as evidenced by markedly decreased phosphorylation levels of both PI3K and Akt (HH vs. Con, *p* < 0.001, Figures 4E–I). Both NBO and HBO treatments effectively reversed this suppression, significantly increasing p-PI3K and p-AKT levels compared to the HH group (HH vs. NBO and HH vs. HBO, *p* < 0.01, Figures 4E–I). Notably, HBO treatment induced a stronger activation of the pathway than NBO (*p* < 0.05 for p-AKT, HBO vs. NBO, Figures 4E,H). In contrast, the total protein expression of PI3K and AKT remained unchanged across all groups, indicating that the observed activation is mediated through post-translational phosphorylation rather than alterations in core protein expression (Figures 4E–I).

Concurrently, we assessed the Nrf-2/HO-1 antioxidant pathway (Figures 4E,J,K). While total Nrf2 protein levels showed a modest increasing trend from the HH group to the oxygen therapy groups, these changes did not reach statistical significance (Figures 4E,J). For HO-1, a significant decrease was observed in the HH group compared to the Con group (*p* < 0.01, Figures 4E,K). However, neither NBO nor HBO treatment resulted in a statistically significant increase in HO-1 expression compared with the HH group (Figures 4E,K).

These findings indicate that, under the present experimental conditions, oxygen therapy primarily influenced hypoxia-induced brain injury through modulation of PI3K–AKT signaling pathways. The lack of significant changes in total Nrf2 protein abundance suggests that Nrf2-mediated responses may involve regulatory mechanisms such as nuclear translocation rather than large alterations in total protein levels.

### 3.4 HBO attenuates neuroinflammation by suppressing the TLR4–NF-κB pathway

**Figure 5:**
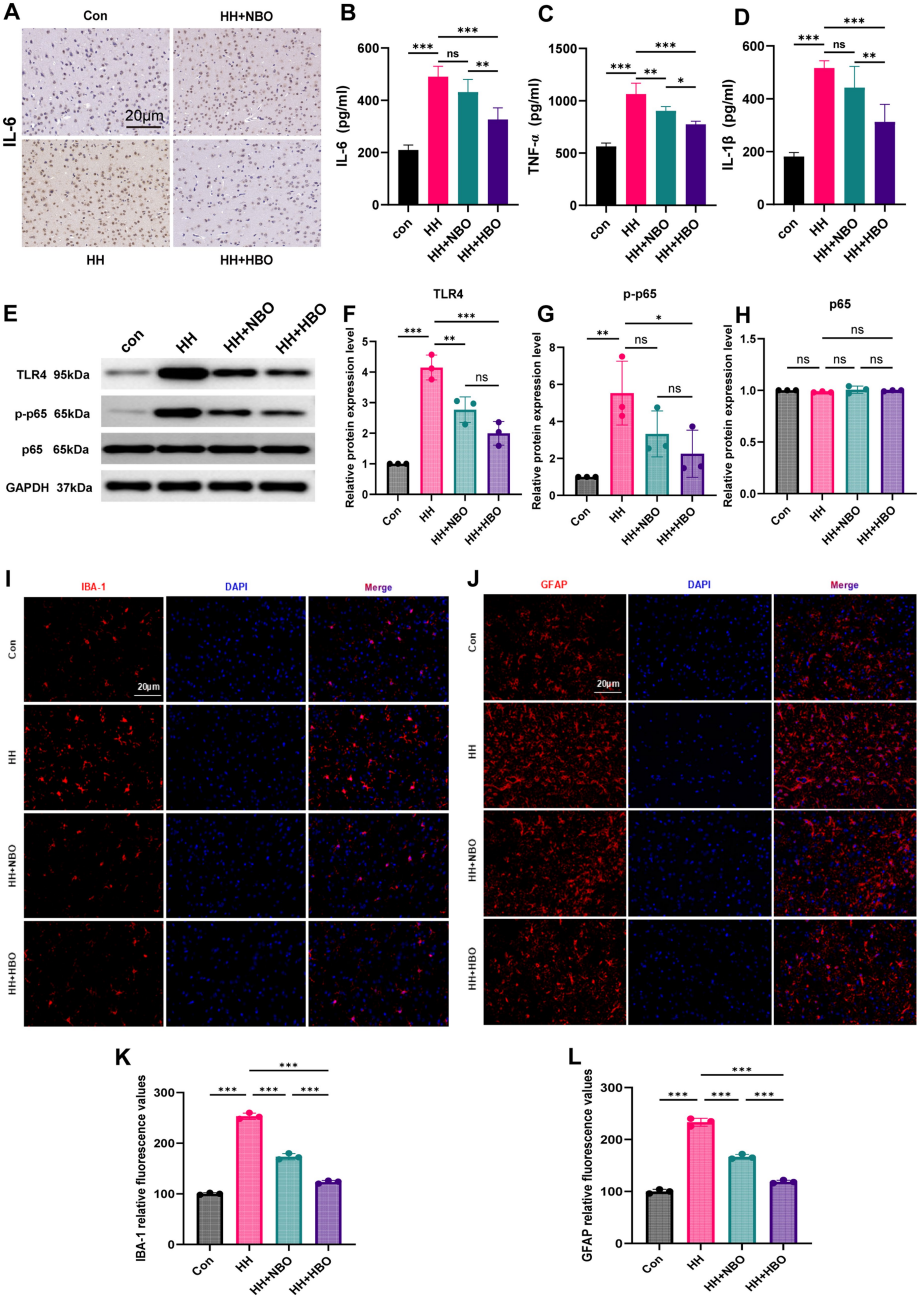
HBO alleviates neuroinflammation by inhibiting the TLR4–NF-κB signaling pathway. **(A)** Representative images of IL-6 immunohistochemical staining in brain tissues across the four groups. Scale bar, 20 μm (*n* = 3 per group). **(B–D)** Serum levels of pro-inflammatory cytokines IL-6 **(B)**, TNF-α **(C)**, and IL-1β **(D)** measured by ELISA. Data are presented as mean ± SDM (*n* = 5 per group). ***p < 0.001, **p < 0.01, *p < 0.05; one-way ANOVA with Tukey’s *post-hoc* test. (E) Representative western blot bands of TLR4, phosphorylated NF-κB p65 (p-p65), total NF-κB p65 (p65), and GAPDH (loading control). **(F–H)** Quantitative analysis of protein expression levels: **(F)** TLR4/GAPDH, **(G)** p-p65/GAPDH, **(H)** p65/GAPDH. Data are mean ± SDM (*n* = 3 independent experiments). ***p < 0.001, **p < 0.01, *p < 0.05; ns, not significant; one-way ANOVA with Tukey’s test. **(I,J)** Fluorescence images of protein expression of IBA-1 and GFAP. Scale bar, 20 m. **(K,L)** Statistical results of protein expression of IBA-1 and GFAP. Bars represent mean ± SD, *n* = 3. ***p < 0.001.

To further investigate the anti-inflammatory effects of HBO, we assessed the expression of key inflammatory mediators and signaling pathways. Immunohistochemical staining revealed markedly elevated IL-6 expression in brain tissues of the HH group, which was visibly reduced following both NBO and HBO treatments, with HBO exhibiting a more pronounced effect (Figure 5A). Consistent with this, ELISA results demonstrated significantly increased serum levels of pro-inflammatory cytokines IL-6, TNF-α, and IL-1β in the HH group (all *p* < 0.001 vs. Con). Both oxygen therapies significantly attenuated these increases; however, HBO was consistently more effective than NBO in reducing cytokine levels (Figures 5B–D).

At the molecular level, Western blot analysis showed significant upregulation of TLR4 protein and increased phosphorylation of NF-κB p65 in the HH group (TLR4: p < 0.001; p-p65: *p* < 0.01 vs. Con), while total p65 levels remained unchanged across groups (Figures 5E–H). HBO treatment significantly suppressed TLR4 expression (*p* < 0.001 vs. HH) and NF-κB activation (*p* < 0.05 vs. HH). Although NBO also reduced TLR4 (*p* < 0.01 vs. HH), its effect on p-p65 was not significant. Notably, the inhibitory effect of HBO on TLR4–NF-κB signaling was significantly stronger than that of NBO (Figures 5F,G).

Furthermore, immunofluorescence staining was performed to visualize microglial (Iba-1+) and astrocytic (GFAP+) activation. Representative images showed increased immunoreactivity for both markers in the HH group, which was markedly reduced by HBO treatment and moderately alleviated by NBO (Figures 5I–L). Quantitative analysis of fluorescence intensity confirmed a significant elevation of Iba-1 and GFAP signals in the HH group, which was robustly attenuated by HBO treatment and moderately by NBO (Figures 5K,L). These results further corroborate the inhibitory effect of HBO on glial activation, consistent with the observed suppression of TLR4/NF-κB signaling and pro-inflammatory cytokines.

In summary, these results indicate that oxygen therapy attenuates hypoxia-induced neuroinflammation, partly through modulation of the TLR4–NF-κB signaling pathway, leading to reduced glial activation and decreased release of pro-inflammatory cytokines. Although both NBO and HBO influenced this inflammatory axis, HBO was associated with a greater degree of pathway modulation, suggesting broader regulatory effects on hypoxia-induced neuroinflammatory responses.

## 4 Discussion

Acute high-altitude exposure induces a hypobaric hypoxic environment that disrupts cerebral homeostasis, triggering a cascade of oxidative stress and neuroinflammatory responses. The current study demonstrates that both normobaric oxygen (NBO) and hyperbaric oxygen (HBO) therapies attenuate hypobaric hypoxia–induced brain injury, likely through coordinated modulation of PI3K–AKT and TLR4–NF-κB signaling pathways.

Hypoxia-induced oxidative stress serves as one of the fundamental determinants of neuronal and glial dysfunction. Reduced oxygen availability disrupts mitochondrial electron transport, leading to the overproduction of reactive oxygen species (ROS), lipid peroxidation, and DNA damage, ultimately initiating apoptosis (Figueroa et al., 2025; Liu Y. et al., 2022). In the current investigation, hyperbaric oxygen (HBO) significantly enhanced antioxidant defense mechanisms, elevating the levels of superoxide dismutase (SOD) and glutathione (GSH) while decreasing malondialdehyde (MDA) and nitric oxide (NO) levels. Normobaric oxygen (NBO) exhibited less prominent effects. Mechanistically, HBO restored the phosphorylation of the phosphatidylinositol 3-kinase (PI3K)–protein kinase B (AKT) pathway without altering the total protein expression, indicating that the post-translational activation of this pathway mediates redox homeostasis and neuronal survival. These findings are in line with reports suggesting that the activation of the PI3K–AKT pathway promotes mitochondrial integrity and restricts ROS-induced apoptosis under hypoxic conditions (He et al., 2019; Liu et al., 2024). Interestingly, although total Nrf2 protein levels did not differ significantly among groups, alterations in its downstream target HO-1 and the oxidative stress markers suggest that redox-responsive pathways were engaged under hypobaric hypoxia. It is well recognized that Nrf2 activity is primarily regulated through dissociation from Keap1 and subsequent translocation from the cytoplasm to the nucleus, rather than through large changes in total protein abundance (Lee et al., 2007; Yamamoto et al., 2018). Therefore, measurement of total Nrf2 protein may not fully reflect the functional activation status of the Nrf2 pathway. Future studies incorporating nuclear-cytoplasmic fractionation or immunofluorescence analysis of Nrf2 localization would provide a more direct assessment of Nrf2 activation under hypoxic stress and oxygen therapy.

Neuroinflammation is a parallel contributor to hypoxia-induced cerebral injury. Activation of TLR4–NF-κB signaling promotes transcription of pro-inflammatory cytokines, including IL-6, TNF-α, and IL-1β, and drives microglial and astrocytic activation (Tao et al., 2024; Wei et al., 2025). HBO effectively suppressed TLR4 expression and NF-κB p65 phosphorylation, attenuating glial activation and downstream cytokine release. This inhibitory effect may synergize with the activation of the PI3K–AKT pathway to suppress excessive inflammatory responses while promoting cell survival. Zhao et al. (2008) confirmed that the PI3K–AKT pathway can negatively regulate the NF-κB signal mediated by Nod2. The mechanism mainly involves AKT in inactivating GSK-3β and reducing the phosphorylation levels of NF-κB p65 subunit at Ser529 and Ser536 sites, thereby inhibiting the transcriptional activity of NF-κB (Zhao et al., 2008). NBO, by contrast, partially inhibited TLR4–NF-κB but did not fully restore pathway homeostasis, explaining its relatively weaker neuroprotective effect.

Transcriptomic analysis revealed that the gene regulatory effects of hyperbaric oxygen (HBO) are not limited to oxidative stress and inflammatory responses. HBO systematically reversed the aberrant expression of multiple hypoxia-induced genes involved in vascular homeostasis. For instance, the expression of aquaporin-4 (Aqp4) and extracellular matrix-related genes exhibited a restorative trend following HBO treatment. AQP4 is a subtype of AQP that is mainly expressed in the foot processes of astrocytes, close to the capillaries, and in the ependymal cells lining the ventricles of the brain (Wang et al., 2025). The possible association between AQP4 and neuroinflammation was first described in neuromyelitis optica (Fukuda and Badaut, 2012). Increasing evidence indicates that AQP4 is involved in neuroinflammation in other brain diseases including brain edema,(Tourdias et al., 2011) experimental autoimmune encephalomyelitis (EAE) (Lennon et al., 2005), and Parkinson’s disease (Li et al., 2011). Li et al. (2011) proposed that AQP4 has an intrinsic pro-inflammatory effect in the central nervous system, which may have broad significance for central nervous system diseases related to neuroinflammation (Lennon et al., 2005). Additionally, in the study by Asai et al. (2013) and Chi et al. (2011), AQP4 was upregulated by pro-inflammatory cytokines in astrocytes. A large number of studies are needed to clarify the relationship between AQP4 regulation and neuroinflammation. Although several studies have separately demonstrated brain inflammation using microglial cells and astrocytes in culture, it is generally believed that neuroinflammation is caused by complex signaling processes involving various brain cells. Aqp4 is a key regulator of water homeostasis at the blood–brain barrier, and its downregulation is directly associated with vasogenic edema, whereas the integrity of the extracellular matrix constrains vascular permeability and inflammatory cell infiltration. Therefore, the normalization of this gene set by HBO suggests that its protective effects may extend to structural repair of the neurovascular unit, beyond merely suppressing inflammatory signaling.

Several limitations warrant consideration. First, the study focused on acute responses to high-altitude exposure and three consecutive oxygen therapy sessions. All molecular and transcriptomic analyses were conducted at a single post-intervention time point; therefore, terms such as “continuously regulated” in the transcriptomic description refer to differential expression status at the time of sampling, rather than implying sustained temporal dynamics. Whether the observed molecular restoration persists, diminishes, or is followed by rebound effects and whether three sessions (HBO) are sufficient to confer durable neuroprotection requires longitudinal profiling over extended post-treatment intervals. Long-term effects of HBO on neuroinflammation, oxidative stress, and functional recovery thus remain to be elucidated. Second, although molecular markers were validated, direct causal relationships between PI3K–AKT activation, TLR4–NF-κB suppression, and neuronal protection require targeted intervention studies. Third, translational applicability to humans needs careful evaluation, given species-specific differences in hypoxia susceptibility and oxygen delivery. In addition, future studies should include both sexes to improve the generalizability of the results. Forth, the relatively small sample size used in several analyses. Although the sample sizes were determined based on commonly accepted practices for exploratory animal studies, larger cohorts will be necessary in future studies to further validate the robustness of these findings.

Whether these acute molecular benefits translate into long-term neurofunctional recovery remains an open question. In related models of cerebral ischemia and traumatic brain injury, early modulation of PI3K–AKT and TLR4–NF-κB signaling has been associated with improved cognitive and motor outcomes at chronic stages (Tao et al., 2024; Zhang J. et al., 2025). By stabilizing the neurovascular unit and attenuating oxidative stress and glial activation at an early phase, HBO may interrupt the self-perpetuating cycle of secondary injury that often leads to progressive neurodegeneration. However, direct evidence linking high-altitude cerebral injury, transient oxygen intervention, and durable functional preservation is currently lacking. Longitudinal studies incorporating behavioral, electrophysiological, and histopathological assessments are therefore warranted to test this hypothesis and to determine whether extended or repeated HBO regimens confer sustained therapeutic benefits.

In summary, both oxygen therapy mitigates high-altitude–induced cerebral injury through coordinated modulation of PI3K–AKT-mediated pro-survival signaling and TLR4–NF-κB-mediated inflammatory responses. This regulation is associated with reduced oxidative stress, attenuated glial activation, and stabilization of transcriptional and neurovascular homeostasis. Compared with NBO, HBO was associated with broader modulation of these molecular pathways. These findings provide mechanistic insight into oxygen therapy in hypobaric hypoxia–induced brain injury and support further investigation of optimized oxygen-based interventions for acute high-altitude cerebral injury.

## 5 Conclusion

In summary, our study demonstrates that both NBO and HBO therapies mitigate hypobaric hypoxia-induced cerebral injury. Oxygen treatment reduced oxidative stress, attenuated neuroinflammatory responses, and modulated key signaling pathways including PI3K–AKT and TLR4–NF-κB. Although the histopathological improvements observed with the two oxygen therapies were comparable, HBO produced broader modulation of molecular signaling pathways associated with oxidative stress and inflammation. No clear evidence of robust activation of the Nrf2/HO-1 pathway was observed at the level of total protein expression, suggesting that Nrf2 regulation under these conditions may involve post-translational mechanisms such as nuclear translocation. These findings provide insight into the molecular mechanisms underlying oxygen therapy in hypobaric hypoxia-induced brain injury and support further investigation of optimized oxygen-based therapeutic strategies for high-altitude cerebral injury.

## Data Availability

The raw RNA-seq data generated in this study have been deposited in the NCBI Sequence Read Archive (SRA, submission ID: SUB16095504) and are associated with the BioProject: PRJNA1447108. The public URL for the project is: https://www.ncbi.nlm.nih.gov/bioproject/PRJNA1447108.
