# Supplementary material for: Transcriptomic profiling and targeted validation reveal molecular mechanisms of oxygen therapy in high-altitude cerebral injury
Source: Front Neurosci. 2026 Apr 13;20:1738756. doi: 10.3389/fnins.2026.1738756 (PMC13111426; doi:10.3389/fnins.2026.1738756)
Supplement: Supplementary file 1 [file Data_Sheet_1.pdf]

Table S1. The differential expression genes (DEGs) in Con vs. HH were clustered using the gene ontology (GO) term annotation.

GO terms	Term Type	P-value	DEGs
blood vessel development	Biological_process	2.44414e-18	85
blood vessel morphogenesis	Biological_process	2.79726e-18	78
animal organ development	Biological_process	1.62229e-17	248
angiogenesis	Biological_process	2.35292e-17	69
vasculature development	Biological_process	3.07247e-17	85
system development	Biological_process	6.34889e-17	282
response to external stimulus	Biological_process	9.85171e-17	200
multicellular organism development	Biological_process	3.46924e-16	315
cell adhesion	Biological_process	9.33935e-16	121
circulatory system development	Biological_process	9.94063e-16	107
extracellular matrix	Cellular_component	3.27195e-22	74
external encapsulating structure	Cellular_component	4.19853e-22	74
extracellular region	Cellular_component	2.32234e-20	195
collagen-containing extracellular matrix	Cellular_component	2.88853e-20	60
cell periphery	Cellular_component	4.12501e-19	397
cell surface	Cellular_component	4.12842e-15	92
intrinsic component of plasma membrane	Cellular_component	1.06246e-13	127
integral component of plasma membrane	Cellular_component	3.40653e-13	121
external side of plasma membrane	Cellular_component	9.62312e-11	49
basal part of cell	Cellular_component	1.07364e-10	39
extracellular matrix structural constituent	Molecular_functions	1.19638e-17	34
extracellular matrix structural constituent conferring tensile strength	Molecular_functions	9.00984e-09	12
protein binding	Molecular_functions	3.35147e-08	1
transporter activity	Molecular_functions	4.707e-08	87
transmembrane transporter activity	Molecular_functions	1.21436e-07	81
calcium ion binding	Molecular_functions	1.25536e-07	60

collagen binding	Molecular_functions	1.53911e-07	15
growth factor binding	Molecular_functions	1.5902e-07	22
extracellular matrix constituent conferring elasticity	Molecular_functions	7.29346e-07	5
cytokine binding	Molecular_functions	7.54436e-07	20
