# Supplementary material for: Transcriptomic profiling and targeted validation reveal molecular mechanisms of oxygen therapy in high-altitude cerebral injury
Source: Front Neurosci. 2026 Apr 13;20:1738756. doi: 10.3389/fnins.2026.1738756 (PMC13111426; doi:10.3389/fnins.2026.1738756)
Supplement: Supplementary file 2 [file Data_Sheet_2.pdf]

Table S2. The differential expression genes (DEGs) in HH vs. NBO were clustered using the gene ontology (GO) term annotation.

GO terms	Term Type	P-value	DEGs
multicellular organism development	Biological_process	7.03938e-22	288
system development	Biological_process	2.56795e-20	253
anatomical structure development	Biological_process	2.63881e-19	313
developmental process	Biological_process	2.02045e-18	329
circulatory system development	Biological_process	7.05612e-18	99
animal organ development	Biological_process	8.19433e-18	215
behavior	Biological_process	4.39109e-17	74
regulation of multicellular organismal process	Biological_process	1.87404e-16	172
tissue development	Biological_process	2.59337e-14	129
tube development	Biological_process	8.4349e-14	89
integral component of plasma membrane	Cellular_component	6.53501e-17	115
intrinsic component of plasma membrane	Cellular_component	1.74893e-16	118
cell periphery	Cellular_component	7.34977e-15	324
extracellular region	Cellular_component	2.61331e-11	144
plasma membrane region	Cellular_component	5.84854e-11	92
cell surface	Cellular_component	6.15171e-11	72
plasma membrane	Cellular_component	2.16003e-10	288
cell projection	Cellular_component	2.38727e-10	143
plasma membrane bounded cell projection	Cellular_component	1.56944e-09	136
extracellular matrix	Cellular_component	2.40622e-09	45
protein binding	Molecular_functions	2.47571e-08	377
signaling receptor binding	Molecular_functions	3.57488e-08	92
oxygen binding	Molecular_functions	8.76578e-07	8

oxygen carrier activity	Molecular_functions	2.31765e-06	6
peptide binding	Molecular_functions	8.52468e-06	26
ion transmembrane transporter activity	Molecular_functions	1.0213e-05	52
protein-containing complex binding	Molecular_functions	2.1736e-05	79
binding	Molecular_functions	3.85242e-05	519
transmembrane transporter activity	Molecular_functions	4.02628e-05	62
growth factor binding	Molecular_functions	4.26544e-05	16
