# Supplementary material for: Transcriptomic profiling and targeted validation reveal molecular mechanisms of oxygen therapy in high-altitude cerebral injury
Source: Front Neurosci. 2026 Apr 13;20:1738756. doi: 10.3389/fnins.2026.1738756 (PMC13111426; doi:10.3389/fnins.2026.1738756)
Supplement: Supplementary file 3 [file Data_Sheet_3.pdf]

Table S3. The differential expression genes (DEGs) in HH vs. HBO were clustered using the gene ontology (GO) term annotation.

GO terms	Term Type	P-value	DEGs
animal organ development	Biological_process	7.43781e-18	219
system development	Biological_process	3.03586e-17	248
multicellular organism development	Biological_process	4.54804e-17	278
anatomical structure development	Biological_process	7.75143e-16	307
tube development	Biological_process	2.30333e-15	94
tube morphogenesis	Biological_process	4.24339e-15	81
developmental process	Biological_process	5.61961e-15	323
blood vessel morphogenesis	Biological_process	5.57244e-14	63
anatomical structure formation involved in morphogenesis	Biological_process	2.19191e-13	91
anatomical structure morphogenesis	Biological_process	4.32427e-13	164
extracellular region	Cellular_component	3.01129e-14	156
extracellular matrix	Cellular_component	1.71793e-10	48
extracellular space	Cellular_component	1.95012e-10	108
external encapsulating structure	Cellular_component	1.97536e-10	48
intrinsic component of plasma membrane	Cellular_component	6.34951e-10	102
integral component of plasma membrane	Cellular_component	1.59489e-09	97
cell periphery	Cellular_component	3.1811e-09	306
apical part of cell	Cellular_component	4.34522e-09	44

apical plasma membrane	Cellular_component	1.20211e-08	38
collagen-containing extracellular matrix	Cellular_component	2.22827e-08	36
extracellular matrix structural constituent	Molecular_functions	2.12259e-09	22
extracellular matrix structural constituent conferring tensile strength	Molecular_functions	1.92327e-08	11
signaling receptor binding	Molecular_functions	5.47361e-08	93
protein binding	Molecular_functions	8.92702e-07	376
oxygen carrier activity	Molecular_functions	2.6539e-06	6
binding	Molecular_functions	3.86113e-06	537
haptoglobin binding	Molecular_functions	6.09897e-06	5
oxygen binding	Molecular_functions	1.37168e-05	7
signaling receptor regulator activity	Molecular_functions	2.90306e-05	40
collagen binding	Molecular_functions	3.23447e-05	11
