# Supplementary material for: Transcriptomic profiling and targeted validation reveal molecular mechanisms of oxygen therapy in high-altitude cerebral injury
Source: Front Neurosci. 2026 Apr 13;20:1738756. doi: 10.3389/fnins.2026.1738756 (PMC13111426; doi:10.3389/fnins.2026.1738756)
Supplement: Supplementary file 4 [file Data_Sheet_4.pdf]

Table S4. The differential expression genes (DEGs) in NBO vs. HBO were clustered using the gene ontology (GO) term annotation.

GO terms	Term Type	P-value	DEGs
synaptic transmission, dopaminergic	Biological_process	8.09236e-06	4
locomotory behavior	Biological_process	4.70903e-05	7
regulation of long-term synaptic potentiation	Biological_process	6.17893e-05	4
long-term synaptic potentiation	Biological_process	6.33389e-05	4
behavior	Biological_process	0.000148376	11
regulation of dopamine uptake involved in synaptic transmission	Biological_process	0.000159173	2
regulation of catecholamine uptake involved in synaptic transmission	Biological_process	0.000159173	2
positive regulation of long-term synaptic potentiation	Biological_process	0.000192228	3
startle response	Biological_process	0.000287677	3
response to amphetamine	Biological_process	0.000409333	3
cilium	Cellular_component	1.43339e-05	12
cell projection	Cellular_component	0.000640263	21
plasma membrane bounded cell projection	Cellular_component	0.00100923	20
MHC class I protein complex	Cellular_component	0.00103144	2
MHC class I peptide loading complex	Cellular_component	0.00185582	2
ciliary membrane	Cellular_component	0.00220248	3

MHC protein complex	Cellular_component	0.00384178	2
Bcl3-Bcl10 complex	Cellular_component	0.00402936	1
radial spoke stalk	Cellular_component	0.00402936	1
Golgi medial cisterna	Cellular_component	0.00453176	2
T cell receptor binding	Molecular_functions	4.11987e-05	3
TAP complex binding	Molecular_functions	0.000706951	2
CD8 receptor binding	Molecular_functions	0.00103144	2
TAP binding	Molecular_functions	0.00121579	2
beta-2-microglobulin binding	Molecular_functions	0.00121579	2
peptide antigen binding	Molecular_functions	0.00235383	2
interleukin-16 binding	Molecular_functions	0.00402936	1
interleukin-16 receptor activity	Molecular_functions	0.00402936	1
apelin receptor activity	Molecular_functions	0.00402936	1
coreceptor activity	Molecular_functions	0.00453176	2
