# Supplementary material for: Transcriptomic profiling and targeted validation reveal molecular mechanisms of oxygen therapy in high-altitude cerebral injury
Source: Front Neurosci. 2026 Apr 13;20:1738756. doi: 10.3389/fnins.2026.1738756 (PMC13111426; doi:10.3389/fnins.2026.1738756)
Supplement: Supplementary file 5 [file Data_Sheet_5.pdf]

Table S5. Summary of the Kyoto Encyclopedia of Genes and Genomes (KEGG) analysis for the top 20 pathways of differentially expressed genes (DEGs) in the comparison between Con and HH.

Pathway	Level 1	P-value	DEGs
Protein digestion and absorption	Organismal Systems	3.65877e-08	20
ECM-receptor interaction	Environmental Information Processing	4.15543e-08	18
PI3K-Akt signaling pathway	Environmental Information Processing	8.65675e-06	35
Complement and coagulation cascades	Organismal Systems	4.83381e-05	14
Hematopoietic cell lineage	Organismal Systems	4.83381e-05	14
Neuroactive ligand-receptor interaction	Environmental Information Processing	0.000109788	32
Malaria	Human Diseases	0.000128433	10
Cytokine-cytokine receptor interaction	Environmental Information Processing	0.000217275	27
Focal adhesion	Cellular Processes	0.000221148	21
African trypanosomiasis	Human Diseases	0.00104025	7
Pathways in cancer	Human Diseases	0.0017736	39
Intestinal immune network for IgA production	Organismal Systems	0.00225272	7
Amoebiasis	Human Diseases	0.00229367	12
Small cell lung cancer	Human Diseases	0.00262869	11
Viral protein interaction with cytokine and cytokine receptor	Environmental Information Processing	0.00286185	11

NOD-like receptor signaling pathway	Organismal Systems	0.00412875	18
Primary immunodeficiency	Human Diseases	0.00461421	6
Epstein-Barr virus infection	Human Diseases	0.00477212	19
Platelet activation	Organismal Systems	0.00882175	12
Vascular smooth muscle contraction	Organismal Systems	0.0102804	13
