# Supplementary material for: Transcriptomic profiling and targeted validation reveal molecular mechanisms of oxygen therapy in high-altitude cerebral injury
Source: Front Neurosci. 2026 Apr 13;20:1738756. doi: 10.3389/fnins.2026.1738756 (PMC13111426; doi:10.3389/fnins.2026.1738756)
Supplement: Supplementary file 6 [file Data_Sheet_6.pdf]

Table S6. Summary of the Kyoto Encyclopedia of Genes and Genomes (KEGG) analysis for the top 20 pathways of differentially expressed genes (DEGs) in the comparison between HH and NBO.

Pathway	Level 1	P-value	DEGs
Malaria	Human Diseases	9.71524e-08	13
ECM-receptor interaction	Environmental Information Processing	5.20283e-06	14
Neuroactive ligand-receptor interaction	Environmental Information Processing	5.32987e-06	32
African trypanosomiasis	Human Diseases	7.39084e-06	9
cAMP signaling pathway	Environmental Information Processing	2.78268e-05	22
TNF signaling pathway	Environmental Information Processing	0.00120204	12
Calcium signaling pathway	Environmental Information Processing	0.00202647	19
Cocaine addiction	Human Diseases	0.00210588	7
Protein digestion and absorption	Organismal Systems	0.00247024	11
Dilated cardiomyopathy	Human Diseases	0.00298572	10
Cholinergic synapse	Organismal Systems	0.00353515	11
MAPK signaling pathway	Environmental Information Processing	0.00415539	21
PI3K-Akt signaling pathway	Environmental Information Processing	0.0052204	24
Rheumatoid arthritis	Human Diseases	0.00530712	9

Th1 and Th2 cell differentiation	Organismal Systems	0.00572673	9
Amoebiasis	Human Diseases	0.00659699	10
Platelet activation	Organismal Systems	0.00761205	11
Morphine addiction	Human Diseases	0.00766662	9
Parathyroid hormone synthesis, secretion and action	Organismal Systems	0.00801276	10
Inflammatory bowel disease	Human Diseases	0.00818817	7
