# Supplementary material for: Transcriptomic profiling and targeted validation reveal molecular mechanisms of oxygen therapy in high-altitude cerebral injury
Source: Front Neurosci. 2026 Apr 13;20:1738756. doi: 10.3389/fnins.2026.1738756 (PMC13111426; doi:10.3389/fnins.2026.1738756)
Supplement: Supplementary file 7 [file Data_Sheet_7.pdf]

Table S7. Summary of the Kyoto Encyclopedia of Genes and Genomes (KEGG) analysis for the top 20 pathways of differentially expressed genes (DEGs) in the comparison between HH and HBO.

Pathway	Level 1	P-value	DEGs
ECM-receptor interaction	Environmental Information Processing	1.8565e-09	18
Malaria	Human Diseases	5.62374e-08	13
Protein digestion and absorption	Organismal Systems	1.39557e-06	16
PI3K-Akt signaling pathway	Environmental Information Processing	1.6851e-05	30
African trypanosomiasis	Human Diseases	0.000317932	7
Neuroactive ligand-receptor interaction	Environmental Information Processing	0.00056381	26
Focal adhesion	Cellular Processes	0.0010329	17
Human papillomavirus infection	Human Diseases	0.00111808	25
AGE-RAGE signaling pathway in diabetic complications	Human Diseases	0.00361809	10
Cushing syndrome	Human Diseases	0.00611746	13
Breast cancer	Human Diseases	0.00726332	12
Cell adhesion molecules	Environmental Information Processing	0.00747304	13
Viral protein interaction with cytokine and cytokine receptor	Environmental Information Processing	0.020868	8
Long-term depression	Organismal Systems	0.0213321	6

Lipid and atherosclerosis	Human Diseases	0.0260873	14
Apelin signaling pathway	Environmental Information Processing	0.0264365	10
Ether lipid metabolism	Metabolism	0.0272106	5
Bile secretion	Organismal Systems	0.0275062	8
Viral myocarditis	Human Diseases	0.027651	7
cAMP signaling pathway	Environmental Information Processing	0.0279446	14
