# Supplementary material for: Transcriptomic profiling and targeted validation reveal molecular mechanisms of oxygen therapy in high-altitude cerebral injury
Source: Front Neurosci. 2026 Apr 13;20:1738756. doi: 10.3389/fnins.2026.1738756 (PMC13111426; doi:10.3389/fnins.2026.1738756)
Supplement: Supplementary file 8 [file Data_Sheet_8.pdf]

Table S8. Summary of the Kyoto Encyclopedia of Genes and Genomes (KEGG) analysis for the top 20 pathways of differentially expressed genes (DEGs) in the comparison between NBO and HBO.

Pathway	Level 1	P-value	DEGs
Antigen processing and presentation	Organismal Systems	0.00210755	3
cAMP signaling pathway	Environmental Information Processing	0.0043607	4
Cocaine addiction	Human Diseases	0.00968007	2
Tryptophan metabolism	Metabolism	0.0112925	2
Allograft rejection	Human Diseases	0.013015	2
Graft-versus-host disease	Human Diseases	0.013015	2
Cell adhesion molecules	Environmental Information Processing	0.0144626	3
Type I diabetes mellitus	Human Diseases	0.0162861	2
Autoimmune thyroid disease	Human Diseases	0.0209516	2
Neuroactive ligand-receptor interaction	Environmental Information Processing	0.023586	4
Alcoholism	Human Diseases	0.025222	3
Viral myocarditis	Human Diseases	0.0261069	2
Th1 and Th2 cell differentiation	Organismal Systems	0.0298016	2
Viral carcinogenesis	Human Diseases	0.0313234	3
Hematopoietic cell lineage	Organismal Systems	0.0330311	2
Human immunodeficiency virus 1 infection	Human Diseases	0.0354047	3
Human T-cell leukemia virus 1 infection	Human Diseases	0.0381404	3
Th17 cell differentiation	Organismal Systems	0.0413116	2
Protein digestion and absorption	Organismal Systems	0.0434932	2

Thiamine metabolism	Metabolism	0.0458271	1
